# Analysis of transcriptome in hickory (*Carya cathayensis*), and uncover the dynamics in the hormonal signaling pathway during graft process

**DOI:** 10.1186/s12864-016-3182-4

**Published:** 2016-11-17

**Authors:** Lingling Qiu, Bo Jiang, Jia Fang, Yike Shen, Zhongxiang Fang, Saravana Kumar RM, Keke Yi, Chenjia Shen, Daoliang Yan, Bingsong Zheng

**Affiliations:** 1 Nurturing Station for the State Key Laboratory of Subtropical Silviculture, Zhejiang A & F University, Linan, Hangzhou, 311300 People’s Republic of China; 2 Center for Cultivation of Subtropical Forest Resources (CCSFR), Zhejiang A & F University, Linan, Hangzhou, 311300 People’s Republic of China; 3 Key Laboratory of Plant Nutrition and Fertilizer, Ministry of Agriculture, Institute of Agricultural Resources and Regional Planning, Chinese Academy of Agricultural Sciences, Beijing, 100081 China; 4 College of Life and Environmental Sciences, Hangzhou Normal University, Hangzhou, 310036 China

**Keywords:** Auxin, Cytokinin, Graft, Hickory, Transcriptome

## Abstract

**Background:**

Hickory (*Carya cathayensis*), a woody plant with high nutritional and economic value, is widely planted in China. Due to its long juvenile phase, grafting is a useful technique for large-scale cultivation of hickory. To reveal the molecular mechanism during the graft process, we sequenced the transcriptomes of graft union in hickory.

**Results:**

In our study, six RNA-seq libraries yielded a total of 83,676,860 clean short reads comprising 4.19 Gb of sequence data. A large number of differentially expressed genes (DEGs) at three time points during the graft process were identified. In detail, 777 DEGs in the 7 d vs 0 d (day after grafting) comparison were classified into 11 enriched Gene Ontology (GO) categories, and 262 DEGs in the 14 d vs 0 d comparison were classified into 15 enriched GO categories. Furthermore, an overview of the PPI network was constructed by these DEGs. In addition, 20 genes related to the auxin-and cytokinin-signaling pathways were identified, and some were validated by qRT-PCR analysis.

**Conclusions:**

Our comprehensive analysis provides basic information on the candidate genes and hormone signaling pathways involved in the graft process in hickory and other woody plants.

**Electronic supplementary material:**

The online version of this article (doi:10.1186/s12864-016-3182-4) contains supplementary material, which is available to authorized users.

## Background

Grafting is a widely used technology in horticulture to aid plant to overcome their limiting factors in growth and reproduction [1]. Many advantages of using grafted plants, such as yield increase, stress tolerance and successive cropping, have been well-studied for several decades [2, 3]. A classical grafting begins with the adhesion of rootstock and scion, followed by the formation of callus tissue at graft interface, and ends with the establishment of vascular connections [4, 5]. After cutting, a mass of callus cells collapse to form a graft junction by cell proliferation [6]. For woody trees, successful grafting is a systemic and biochemical process associated with various hormonal and environmental factors [7, 8].

Homeostasis of several phytohormones involved in callus formation during plant wound responses, plays important roles in the graft process [9–11]. In the model plant *Arabidopsis*, auxin signaling is required for graft union development. Transport activities of vasculature are recovered at 3 d after grafting, and auxin modulates vascular reconnection at an earlier stage [12]. In particular, auxin plays an essential role in vascular development and vein formation, and application of exogenous auxin to callus enhances the formation of xylem and phloem [13, 14]. Similar to *Arabidopsis*, a microarray data from grapevine identifies an auxin influx carrier encoding gene that is up-regulated at the early stage (3 d) after grafting in graft interface zone [15]. At the molecular level, expression of many auxin downstream genes is regulated during the formation of graft union [5, 16]. In addition, the cytokinin-signaling pathway has also been reported to be involved in the early stage of the graft process. In tomato, rootstock-mediated changes in the status and concentration of zeatin, one of the major cytokinins in plants, are correlated with leaf senescence and crop productivity under salinity [11]. In cotton, a grafting experiment indicated important effects of rootstock on leaf senescence by regulating endogenous cytokinin [17]. In grapevine, genes related to cytokinin biosynthesis are highly enriched in the induced gene list at 28 d after grafting [15].

Hickory (*Carya cathayensis* Sarg.), a popular nut tree, is widely distributed and broadly planted in Tianmu Mountain, Zhejiang Province, China, due to its nutritional and commercial value. Limited to its 10-year juvenile phase, the yield stagnation of hickory has been a serious problem for many years. Grafting is an effective approach to reduce the reproductive cycle of hickory trees, and makes the cultivation of hickory on a large area possible [18]. The healing process between two partners (rootstock and scion) occurs in the graft union and is a key step of grafting in plants [19]. In recent years, 49 unigenes associated with the graft process were identified in the graft union of hickory [16]. Furthermore, the microRNA expression patterns during the graft process were also identified in hickory [18].

To reveal the molecular mechanism of early responses during the graft process, we sequenced the transcriptome of the graft union in hickory at different time points, and the kinetics of the expression patterns of genes related to the auxin-and cytokinin-signaling pathway related genes in hickory were also revealed. Analysis of the gene expression responses to grafting in hickory will aid understanding of the involvement of hormonal signaling in the grafting process for woody plants in general.

## Methods

### Experiment design, plant materials and RNA extraction

Hickory (*Carya cathayensis* Sarg.) trees were planted at a green house in the campus of Zhejiang Forestry University, Lin’an, China. Hickory materials were collected from the graft unions (the stem segment comprises of the swollen part of the rootstock and the scion) at 7 and 14 days after grafting. The first day of grafting was used as the basis of sampling time. In detail, the time point 7 d was chose for investigating the early response of the transcriptome during grafting process; while the time point 14 d was chose for investigating the differential expressed genes during the callus formation. The samples from rootstock (2 years old) and scion (1 year old) before grafting were used as controls. Total RNA was extracted from different plant samples using RNeasy plant mini kits (Qiagen, Hilden, Germany) following the manufacturer’s protocol. RNA contamination was checked using a NanoPhotometer® spectrophotometer (Implem, CA, USA) and removed by 1% agarose gels electrophoresis.

### Construction of cDNA library for digital gene expression (DGE) analysis

In total, 3 μg of RNA per sample was used for cDNA library preparation. Sequencing libraries were constructed by NEBNext® Ultra^TM^ RNA Library Prep Kit for Illumina® (NEB, Ipswich, MA, USA) following the protocol. In summary, mRNA was isolated from total RNA using poly-T oligo-attached magnetic beads. Fragmentation was carried out in NEBNext First Strand Synthesis Reaction Buffer at an elevated temperature. First-strand cDNA was synthesized by a random primer and M-MuLV Reverse Transcriptase (RNase H^−^) and second-strand cDNA was synthesized by DNA Polymerase I and RNase H. Remaining overhangs were blunted using exonuclease/polymerases. NEBNext adaptor with hairpin loop structure was ligated to adenylated 3’-ends of DNA fragments for hybridization. To preferentially select 150–200 bp cDNA fragments, the library fragments were purified using the AMPure XP system (Beckman Coulter, Beverly, USA). Then, 3 μl of USER Enzyme (NEB, Ipswich, MA, USA) was added to the size-selected and adaptor-ligated cDNA fragments at 37 °C for 15 min followed by 5 min of 95 °C treatment before the PCR process. Then PCR was carried out using Phusion High-Fidelity DNA polymerase kit (NEB, Ipswich, MA, USA), Universal primers and Index (X) Primer. Finally, PCR products were purified by AMPure XP system (Beckman Coulter, Beverly, USA) and the quality of library was evaluated by Agilent Bioanalyzer 2100 system (Agilent, Santa Clara, USA).

### Sequencing, quality control and *de novo* assembly

The hickory sample library preparations were sequenced on an Illumina HiSeq 2000–/2500 platform, and raw reads were exported in fastq format. The reads of low quality were removed to generate clean reads, which were used for further analyses. The clean reads were then *de novo* assembled using the Trinity assembly program (Release 2012–10–05).

### Sequence annotation

Functional annotation of unigenes was carried out using BLASTing with an *E*-value threshold of 10^−5^ to various protein databases, including Swiss-Prot protein, NCBI non-redundant (NR), and Kyoto Encyclopedia of Genes and Genomes (KEGG) databases. Sequences with highest similarities were retrieved for further analysis. KEGG was used to annotate the metabolic pathway, and Blast2GO was used to Gene Ontology (GO) classifications.

### Differential expression analysis

The alignment software, Bowtie 0.12.8, was used to map the reads to the transcriptome. The expression level of unigene was calculated basing on its Reads Per kb per Million reads (RPKM), which is the number of mapped clean reads for each unigene. Two independent biological replicates were performed in the experiment. Differential expression analysis was carried out using the DESeq R package (version, 1.10.1). A negative binomial distribution-based model was used for DESeq to perform statistical routines for determining differential expression in DGE data. To control the false discovery rate, the *P* values of results were adjusted by the Benjamini and Hochberg’s method. Genes with an adjusted *P*-value < 0.05 were assigned as differentially expressed. Normalized gene transcript abundance value was calculated by dividing each RPKM value by their average value cross all samples and then taking its log_2_ as the base. All the differential expressed genes were grouped into 14 clusters using MultiExperiment Viewer (MeV) (version 4.9.0) by a K-means method. Pearson’s correlation was used as the default distance metric in MeV software for similarity distance computing.

### Enrichment analysis of GO enrichment and KEGG pathway

GO enrichment analysis was carried out using the GOseq R packages based on Wallenius non-central hyper-geometric distribution. Then, the significantly enriched GO terms were analyzed using hyper geometric test with *P*-value ≤ 0.01. KEGG is a database resource (http://www.genome.jp/kegg/) for studying of high-level bio-functions and utilities of biological system, especially large-scale sequence datasets generated by genome sequencing and other high-throughput experimental technologies. Here, KOBAS software was used to test the statistical enrichment of DEGs in KEGG pathways. GO enrichments and KEGG pathway enrichments were compared within up-regulated and down-regulated unigenes.

### Protein-protein interaction (PPI) analysis

All identified amino acid sequences of the DEGs were searched against the online STRING database (version 9.1, http://string-db.org/) for PPI prediction. The interactions between the proteins belonging to the searched data set were selected, excluding external candidates. The STRING defines a metric called ‘confidence score’ to define the interaction confidence; we selected all interactions with a confidence score higher than 0.7. Then, the PPIs of these DEGs were visualized in Cytoscape (Version, 3.2.1).

### Search for homologous genes

Searches for hormone pathway genes in other plant species were performed using the NCBI (http://www.ncbi.nlm.nih.gov/), TAIR (http://www.arabidopsis.org/), RGAP (http://rice.plantbiology.msu.edu/), and Phytozome (http://www.phytozome.net/, Version, 10.1) databases. Subsequently, searches for homologous hormone-related genes in hickory were performed from our transcriptome data using the BLAST program. Motifs and domains of homologous hickory genes were identified using the databases, including Panther (http://www.pantherdb.org/) and Pfam (http://pfam.xfam.org/).

### Real-time PCR (qRT-PCR) validation

The qRT-PCR primers sequences were designed by Primer Premier 5 software (Premier Biosoft International, Palo Alto, CA, USA). The hickory *Actin* gene was used to calculate the relative fold-differences based on comparative cycle threshold (2^-ΔΔ*Ct*^) values. The qRT-PCR procedure was as follows: 1 μL of a 1/10 dilution of cDNA in H_2_O was added to 5 μL of 2× SYBR® Green buffer, 0.1 μM of each primer, and H_2_O to a final volume of 10 μL. Differences between two samples were calculated by one-way analysis of ANOVA with Student’s *t*-test at a significance level of 0.05 in Excel software. All expression analysis was carried out for seven biological repeats and the average values of seven repeats values were shown in figures.

## Results

### Illumina sequencing, assembly and unigene annotation

**Fig. 1:**
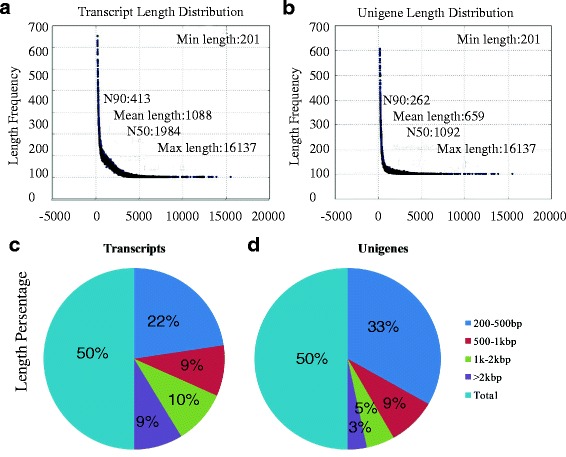
Length distribution of assembled hickory transcripts and unigenes. All clean reads from each sample were combined and resulted in 160,638 transcripts and 89,633 unigenes. **a**, **c** The length distribution of assembled transcripts in hickory. **b**, **d** The length distribution of assembled unigenes in hickory

For RNA-seq, total RNA isolated from two biological replicates for each sample was subjected to cDNA library preparation to generate a broad survey of transcripts associated with the graft process in hickory. Raw Illumina sequencing reads were qualified and adapter-trimmed to yield a total of 83,676,860 clean short reads comprising 4.19 Gb of sequence data from all the complementary DNA libraries (Additional file 1). The sequence generated from Trinity software was used as the reference transcriptome [20]. Additionally, all the reads obtained from hickory at three grafting stages were assembled using Trinity, and then the low complexity and low-quality reads were filtered out, generating 160,638 transcripts (N50: 1,984) with a mean length of 1,088 bp. For each sample, about 93% reads were mapped to the reference transcriptome by RSEM software with default parameters [21]. Clustering resulted in 89,633 unigenes (N50: 1,092) with the mean length of 659 bp (Fig. 1a, b). We also statistically determined that there were 30,967 transcripts (10%) in the length range of 1,000 to 2,000 bp and 27,983 (9%) with length > 2,000 bp; while there were 8,821 unigenes (5%) in the range of 1,000–2,000 bp and 5,991 (3%) with length > 2,000 bp (Fig. 1c, d).

To functionally annotate the assembled hickory unigenes, we compared their sequences against various protein databases using BLASTX. In total, there were 37,084 unigenes (41.37%) annotated in the Nr database, 17,990 (20.07%) in the Nt database, 7,010 (7.82%) in the KEGG database, 25,438 (28.38%) in the SwissProt database, 25,582 (28.54%) in the PFAM database, 29,947 (33.41%) in the GO database, and 13,735 (15.32%) in the KOG database (Additional file 2). Based on these annotations, we identified a total of 41,603 (46.41%) unigenes annotated in at least one database, suggesting that a relatively large portion of hickory unigenes had no hits to any known proteins in the selected databases.

### Classification of enriched GO and KEGG terms

We further assigned GO terms to hickory unigenes. A total of 29,947 unigenes (33.41%) could be assigned to at least one GO term, and detailed information on the classification of enriched GO is listed in Additional file 3. Within the biological process category, the most highly represented terms were ‘cellular process’ and ‘metabolic process’. Within the molecular function category, ‘catalytic activity’ and ‘binding’ were the two most abundant terms. The most enriched terms within the cellular component category were ‘Organelle’, ‘cell’ and ‘cell part’ (Additional file 3).

To further reveal the involvement of metabolic pathways in graft process, we predicted the KEGG pathways represented by all assembled unigenes. A total of 12,034 unigenes were predicted in 248 signaling and metabolic pathways, including pathways related to cellular process, environmental information processing, genetic information processing, metabolism, and organismal systems (Additional file 4). Interestingly, the most enriched KEGG pathways included those related to metabolic pathways, such as amino acid metabolism (1106 unigenes), carbohydrate metabolism (1721 unigenes), and energy metabolism (1461 unigenes) (Additional file 5).

### Analysis of DEGs during the hickory graft process

**Fig. 2:**
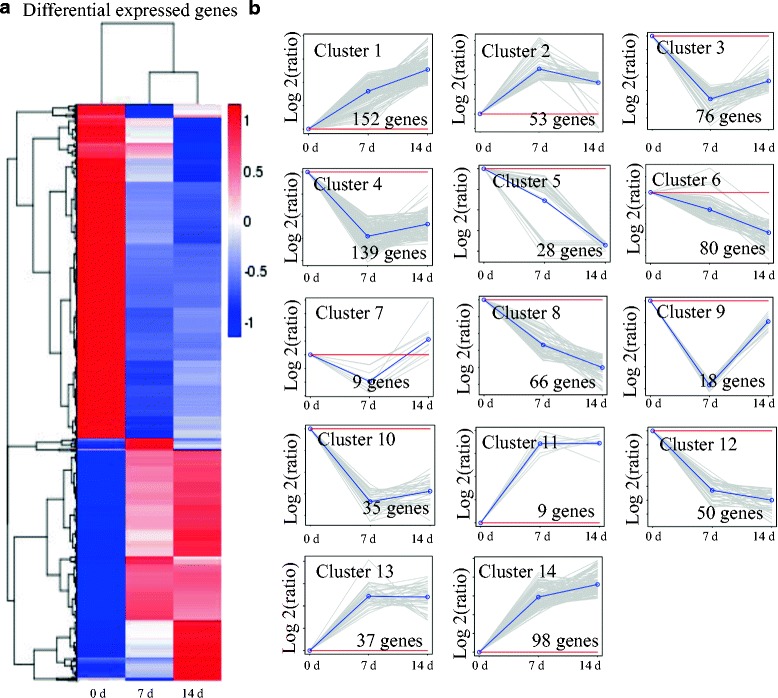
Expression profiles of the differentially expressed transcripts during the graft process in hickory. **a** Heat map for cluster analysis of the differentially expressed unigenes by K-means method. Red indicates up-regulated genes and blue indicates down-regulated genes. **b** MeV cluster analysis of differentially expressed genes from the gene expression profiles

RNA-Seq analysis of the gene expression during the graft process in hickory was conducted at time points 0, 7 and 14 d. RNA-Seq data were processed to calculate RPKM values, which are normalized indicators for comparing the transcript levels of each unigene between different samples. A total of 850 significantly DEGs were identified and analyzed using criteria of two-fold differences and padj < 0.05 (Fig. 2a). To show the major trends and the major transitional states during the graft process in hickory (0, 7, and 14 d), all 850 DEGs were assigned to 14 clusters by K-means method. Among these up-regulated gene clusters, clusters 1 and 14 showed a similar pattern of genes were up-regulated at different time points and reaching their peak levels at time point 14 D; The genes of clusters 2, 11, and 13 were also up-regulated during the graft process and reached their peak levels at time point 7 d. Clusters 3–6, 8, 10 and 12 were significantly down-regulated. The genes of clusters 7 and 9 were down-regulated at time point 7 d and increased at time point 14 d (Fig. 2b).

**Fig. 3:**
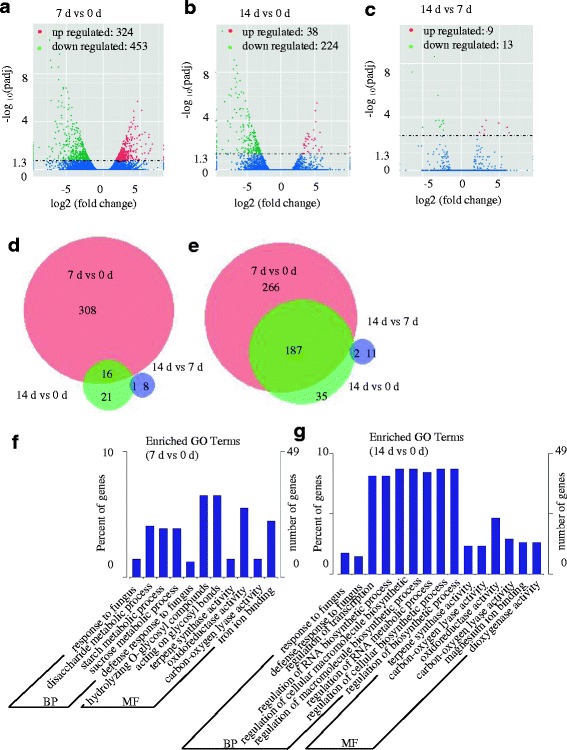
Analysis of the differentially expressed unigenes (DEGs) during the graft process in hickory. **a**-**c**) Volcanoplots of the DEGs in different comparisons. **d**, **e** VennDiagrams of the DEGs in different comparisons. **f** Gene Ontology (GO) classifications of DEGs in the Cc_7 D vs Cc_0 D comparison. **g** Gene Ontology (GO) classifications of DEGs in the Cc_14 D vs Cc_0 D comparison

We then identified highly genes that were differentially expressed between any two of the three time points during the graft process. Based on the same criteria (two-fold and padj < 0.05), we identified 777 unigenes that were significantly differently expressed at time point 7 d compared with control (time point 0 d); of these, 324 unigenes were up-regulated and 453 unigenes down-regulated (Fig. 3a). This was a greater number than the differentially expressed unigenes between time points 14 d and 0 d, for which a total of 262 unigenes were identified, with 38 up-regulated and 224 down-regulated (Fig. 3b). A small number of differentially expressed unigenes were identified between time points 14 d and 7 d: nine unigenes up-regulated and 13 down-regulated (Fig. 3c). We compared three sets of transcriptome data from different time points. Interestingly, most of the differentially expressed unigenes in the comparison between 7 and 0 d were independent of those in the comparison between 14 d and 0 d. In detail, only 16 unigenes were up-regulated both in the comparisons between 7 d and 0 d, and 14 d and 0 d; while 187 unigenes were down-regulated in both comparisons (Fig. 3d, e).

Furthermore, to demonstrate useful information concerning the DEGs during the graft process, we analyzed the GO terms represented by these genes. In total, 11 enriched GO terms were identified within the DEGs between 7 d and 0 d; while 15 enriched GO terms were identified between 14 d and 0 d. GO term enrichment analysis indicated that genes involved in various biological processes and molecular functions such as response to fungus, defense response to fungus, terpene synthase activity, oxidoreductase activity and carbon-oxygen lyase activity were significantly enriched in DEGs both in both the 7 and 0 d and 14 d and 0 d comparisons (Fig. 3e, f).

### PPI network of DEGs

**Fig. 4:**
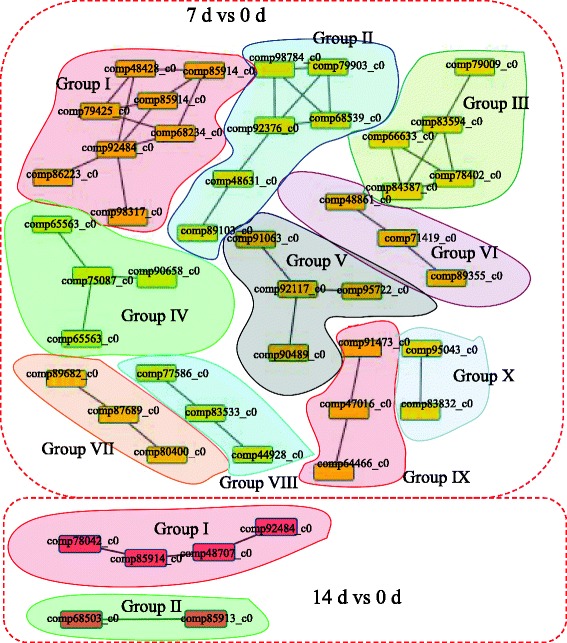
Interaction network of the differential expressed gene encoding proteins analyzed by Cytoscape software (version 3.0.1). The interaction networks of differential expressed genes in Cc_7 D vs Cc_0 D and Cc_14 D vs Cc_0 D comparisons are shown in different colors, respectively

To further investigate the biological processes involved in the grafting process of hickory, we analyzed the PPIs among the 850 identified DEGs. The PPI network for hickory graft process had 47 proteins as nodes, connected by a number of identified direct physical interactions obtained from the STRING database. A high quality image was constructed as an overview of the PPI network, with differentially enriched interaction groups indicated by different colors (Fig. 4 and Additional file 6).

It is noteworthy that 10 clusters were identified within the DEGs in the comparison between 7 and 0 d; however, only two clusters were identified within the DEGs in the comparison 14 d and 0 d. In detail, the largest cluster (cluster I) consisted of eight proteins related to the biosynthesis and degradation of lignin. The second largest cluster (cluster II) consisted of six proteins, which are molecular chaperone involved in R gene-mediated disease resistance. Five ribosomal proteins were grouped into the third largest cluster (cluster III). Two PPIs were identified from the DEGs in the comparison between 14 d and 0 d: cluster I (involved in lignin biosynthesis) and cluster II (transcriptional repressor involved in abiotic stress responses). Interestingly, the proteins associated with lignin biosynthesis and degradation showed significant differences in both comparisons between 7 and 0 d and between 14 d and 0 d.

### Expression of auxin signaling pathway and cytokinin signaling pathway genes during the graft process

**Fig. 5:**
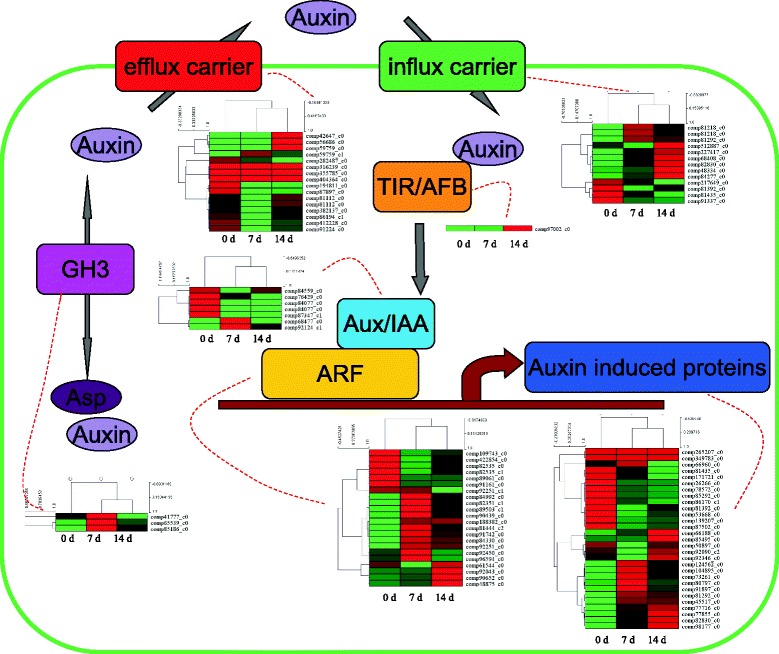
Transcript abundance changes of auxin signaling-related genes in hickory during graft process

Expression of genes of the auxin and cytokinin signaling pathways were analyzed to reveal the involvements of these two important hormonal signaling pathways in the graft process in hickory [22]. Comparison of the transcript abundances of auxin transport, metabolism, signaling pathway, and downstream induced genes revealed a conserved response during the graft process (Fig. 5). The transcription level of most auxin transporter encoding genes was changed significantly during the graft process. For auxin efflux carriers, several unigenes (comp42647_c0, comp56686_c0 and comp59759_c0) were up-regulated at time point 14 d; while several other unigenes (comp282487_c0, comp194811_c0 and comp87897_c0) were down-regulated at time points 7 d and 14 d. For auxin influx carriers, most unigenes were induced and four unigenes (comp217649_c0, comp81392_c0, comp81435_c0 and comp91337_c0) were greatly down-regulated during the graft process. Only one TIR/AFB encoding gene was identified in the hickory transcriptome data, and its expression level was induced at time point 14 d. Three indole-3-acetic acid-amido synthetase genes were identified: comp41777_c0, comp65539_c0 and comp85186_c0. Interestingly, these three unigenes displayed highest expression level at the early stage (7 d), and then decreased at time point 14 d. Many unigene sequences were appraised as auxin response family genes (Aux/IAA and ARF families), and these genes showed a diversity of expression pattern during the graft process (Fig. 5 and Additional file 7).

**Fig. 6:**
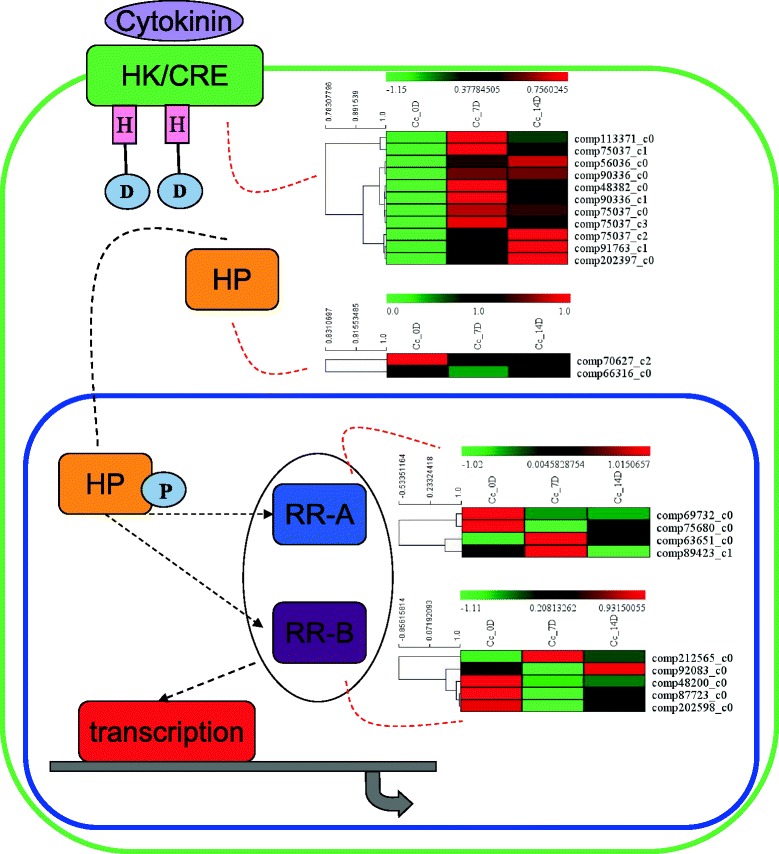
Transcript abundance changes of cytokinin signaling-related genes in hickory during graft process

Furthermore, genes related to cytokinin signaling pathway were also identified from the hickory transcriptome data. It is noteworthy that all 11 hickory AHK/CRE genes were up-regulated during the graft process. Expression of some AHK/CRE genes (comp113371_c0, comp75037_c0, comp48382_c0 and comp90336_c0) peaked at time point 7 d, and then declined slightly at time point 14 d. Two RR-A genes, comp69732_c0 and comp75680_c0, were reduced during the graft process; while another two RR-A genes, comp63651 _c0 and comp89423_c1, were induced at time point 7 d, and then dropped down to the base level (0 d). For RR-B genes, one unigene (comp212565_c0) was up-regulated at time point 7 d, another unigene (comp92083_c0) was up-regulated at time point 14 d, and the rest ones (comp48200_c0, comp87723_c0 and comp202598_c0) were down-regulated at time points both 7 d and 14 d (Fig. 6 and Additional file 8). Furthermore, the phylogenetic data of auxin signaling pathway and cytokinin signaling pathway genes was showed in Additional file 9.

### QRT-PCR validation of the expression level of several unigenes from RNA-seq data

**Fig. 7:**
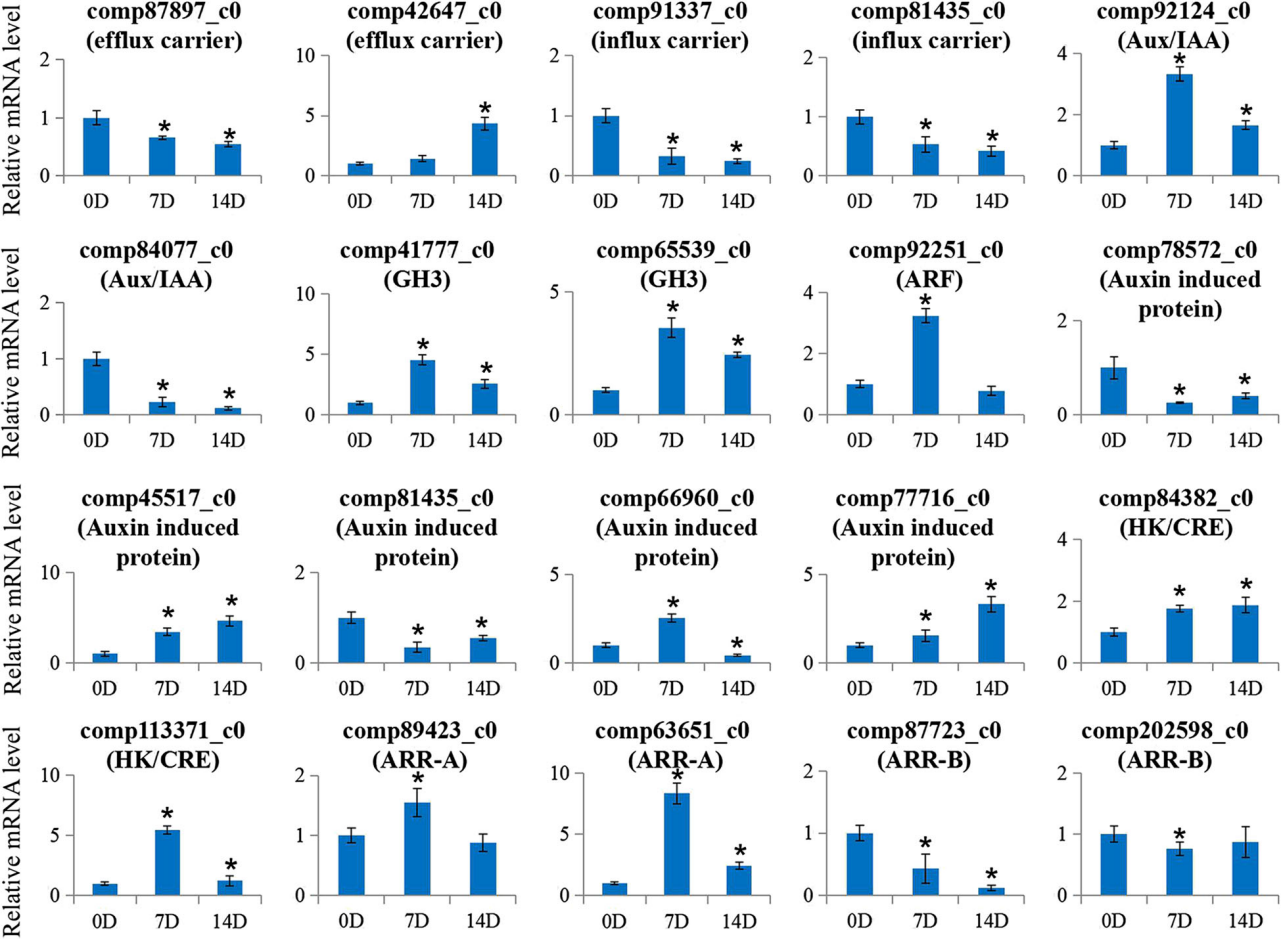
Real-time quantitative PCR validation of several selected hormone-related genes in hickory during graft process. Total RNA was extracted from the scion and rootstock of the grafted hickory at different time points after grafting. The histogram shows the relative expression level of these genes with respect to the ACTIN in hickory. The data were analyzed by three independent repeats, and standard deviations were shown with error bars. Significant differences in expression level were indicated by “*”

To verify the DEGs related to hormone signaling that were identified using RNA-Seq, we performed qRT-PCR assays with independently samples collected from graft unions during the different grafting stages (0, 7 and 14 d). We selected 20 unigenes from auxin- and cytokinin-signaling pathways, including two efflux carriers, two influx carriers, two Aux/IAAs, two GH3s, one ARF, five auxin induced proteins, two HK/CREs, two ARR-As and two ARR-Bs, to validate the RNA-Seq data. The expression levels of these selected genes were basically consistent with RNA-Seq results (Fig. 7). The primer sequences are listed in Additional file 10.

## Discussion

Despite being a famous nut from a woody plant, increasing yield has always been limited by a long vegetative period before the reproductive stage of hickory [23]. Therefore, grafting is widely used for hickory cultivation in South China. Studying the dynamics of transcriptomes is useful for exploring the mechanism of transcriptional regulation during grafting process [24].

In our study, six RNA-seq libraries yielded a total of 83,676,860 clean short reads comprising 4.19 Gb of sequence data (Additional file 11: Table S1), smaller than that in the previously reported woody plants: such as longan (*Dimocarpus longan* L.), lilac (*Syringa oblata* L.), and Siberian apricot (*Prunus sibirica* L.) [25–27]. It is worth to mention that a total of 89,633 unigenes were obtained from the clean reads, which is similar to longan (68,905 unigenes), Siberian apricot (124,070 unigenes), and lilac (104,691 unigenes); additionally, the average length of unigenes in hickory (659 bp) was also on the same level to longan (448 bp), Siberian apricot (830 bp), and lilac (853 bp) [25–27]. These results demonstrated that our sequencing data could be used for gene discovery in the non-model woody tree, hickory.

Based on the enriched GO terms, many DEGs in the comparison between 7 d and 0 d are presumably related to carbohydrate and energy metabolism, such as disaccharide, starch, and sucrose metabolic processes [28] (Fig. 3f). In the 14 d and 0 d comparison, a large number of DEGs associated to macromolecule biosynthetic process were identified in the enriched GO terms (Fig. 3g). Adhesion between the cells from scion and rootstock is enhanced by binding material, composed of pectin, carbohydrate, protein and fatty acids [29]. Activation and induction of metabolic activities may contribute to nutrient transportation for the biosynthesis of the binding material [8]. Interestingly, the ‘response to fungus’ GO term was identified in both comparisons between 7 d and 0 d and between 14 d and 0 d, suggesting an occurrence of quick defense response during the graft process [30]. Furthermore, oxidative stress in graft interfaces has been reported in various species (Fernandez-Garcia et al., 2004; Irisarri et al., 2015). In tomato, the activities of many antioxidant enzymes, such as superoxide dismutase, catalase and ascorbate peroxidase, were induced in the graft unions (Muneer et al., 2016). A number of differential expressed antioxidant genes were identified in the heterograft of pear/quince combinations (Irisarri et al., 2015). In our study, the ‘oxidoreductase activity’ GO term was identified in both comparisons between 7 d and 0 d and between 14 d and 0 d, indicating that grafting generally triggers antioxidant defense systems in hickory.

Callus tissue formation at the graft union is the first and a basic response to grafting, and lack of callus formation is a major cause of grafting failure [7]. In addition, the callus cells differentiating into vascular tissue to re-connect the xylem and phloem at the graft junction is also thought to be a key step for a successful grafting [6]. Auxin and cytokinin are two major hormones involved in vascular differentiation and reconnection, and so we tried to understand their roles in hickory grafting [31]. Mutations in several auxin signaling genes including *ARF5*, *ARF6*, *ARF8* and *IAA12* perturb vascular patterning and reduce cell division in the pith cells after cutting [32, 33]. In hickory, a large number of *ARF* and *Aux/IAA* genes were identified. The changes in their expression patterns indicated a key role of auxin signaling in cell division and vascular reestablishment during the grafting process. In model plants, a block in auxin transport at the graft junction could cause auxin accumulation in the scion, increasing xylem differentiation [8]. Another study reported that vasculature transport activities were recovered at 3 days after grafting and that auxin regulated the vascular reconnection at 2 days after grafting [12]. Many efflux carriers in hickory, such as comp87897_c0 and comp42647_c0, were significant changed by grafting, suggesting that increasing auxin promotes callus formation from xylem pole pericycle cells [8]. Besides, the expression of three *GH3* genes, comp41777_c0, comp65539_c0 and comp85186_c0, also showed responses to grafting in hickory. In grape, the different kinetic of IAA-Asp accumulation at the grafting stages was associated to the expression pattern of *GH3* gene, namely *VviGH3-21* [34]. This shift in IAA-Asp accumulation may play an important role in the grafting process of hickory.

Additionally, there is accumulating evidence that cytokinin also participates in vascular differentiation [31]. Thus, we analyzed the expression changes in genes related to cytokinin signaling to test whether cytokinin signaling contributed to the formation of graft union in hickory. Cytokinin receptors histidine kinases (HK/CREs), a major component of the cytokinin signaling, trigger a phosphorelay by binding to cytokinin [35]. The expression of most HK/CRE homologous genes in hickory was largely up-regulated at time point 7 d, indicating an activation of cytokinin signaling during the graft process in hickory. There are 23 functional response regulators (RRs) have been identified in *Arabidopsis*; however, only four RR-A type and five RR-B type genes were annotated by our transcriptome data [35–37]. Interestingly, two RR-A homolog, comp63651_c0 and comp89423, and one RR-B homolog, comp212565_c0, were up-regulated at time point 7 d, and then recovered to the control level (0 d) at time point 14 d in hickory, suggesting their expression was specific to the early stage of graft union formation.

## Conclusions

In our study, three independent cDNA libraries from hickory at 0, 7, 14 d post-grafting were constructed and sequenced. Many of DEGs were identified in hickory during the graft process. Transcription dynamics of grafting response genes and their related major biological functions were grouped into different GO and KEGG categories. Furthermore, the expression of genes related to auxin-and cytokinin-signaling pathways was analyzed in hickory, and some were validated by qRT-PCR analysis. Identification and analysis of these auxin and cytokinin-related genes will aid us to understand the complexity of hormones during the graft process in plants.

## Additional files

Additional file 1: The QC summary of clean data from different samples. (XLSX 9 kb)
Additional file 2: Annotation statistical proportion of unigenes. (XLSX 9 kb)
Additional file 3: The information of the classification of the enriched GO. (XLSX 10 kb)
Additional file 4: The classification of the GOs. (PDF 205 kb)
Additional file 5: The information of the classification of the KEGGs. (XLSX 9 kb)
Additional file 6: The classification of the KEGGs. (PDF 181 kb)
Additional file 7: The expression level of significantly differential expressed genes. (XLSX 58 kb)
Additional file 8: The expression level of auxin signaling pathway related genes. (XLSX 15 kb)
Additional file 9: The expression level of cytokinin signaling pathway related genes. (XLSX 11 kb)
Additional file 10: The phylogenetic data of auxin signaling pathway and cytokinin signaling pathway genes. (XLSX 28 kb)
Additional file 11: The primer sequences of ten selected genes. (XLSX 10 kb)

## Abbreviations

ARF: Auxin response factor
Aux/IAA: Auxin/indole-3-acetic acid
BLAST: Basic local alignment search tool
DEG: Differentially expressed gene
DGE: Digital gene expression
GO: Gene ontology
HK: Histidine kinase
KEGG: Kyoto encyclopedia of genes and genomes
MeV: MultiExperiment viewer
PCR: Polymerase chain reaction
PPI: Protein-protein interaction
qRT-PCR: Real-time polymerase chain reaction
RPKM: Reads Per Kb per Million reads
RR: Response regulator
