# Supplementary figures and images for: Analysis of transcriptome in hickory (Carya cathayensis), and uncover the dynamics in the hormonal signaling pathway during graft process
Source: BMC Genomics. 2016 Nov 17;17:935. doi: 10.1186/s12864-016-3182-4 (PMC5114764; doi:10.1186/s12864-016-3182-4)

Figure S1 The information of gene function classification (GO) analysis

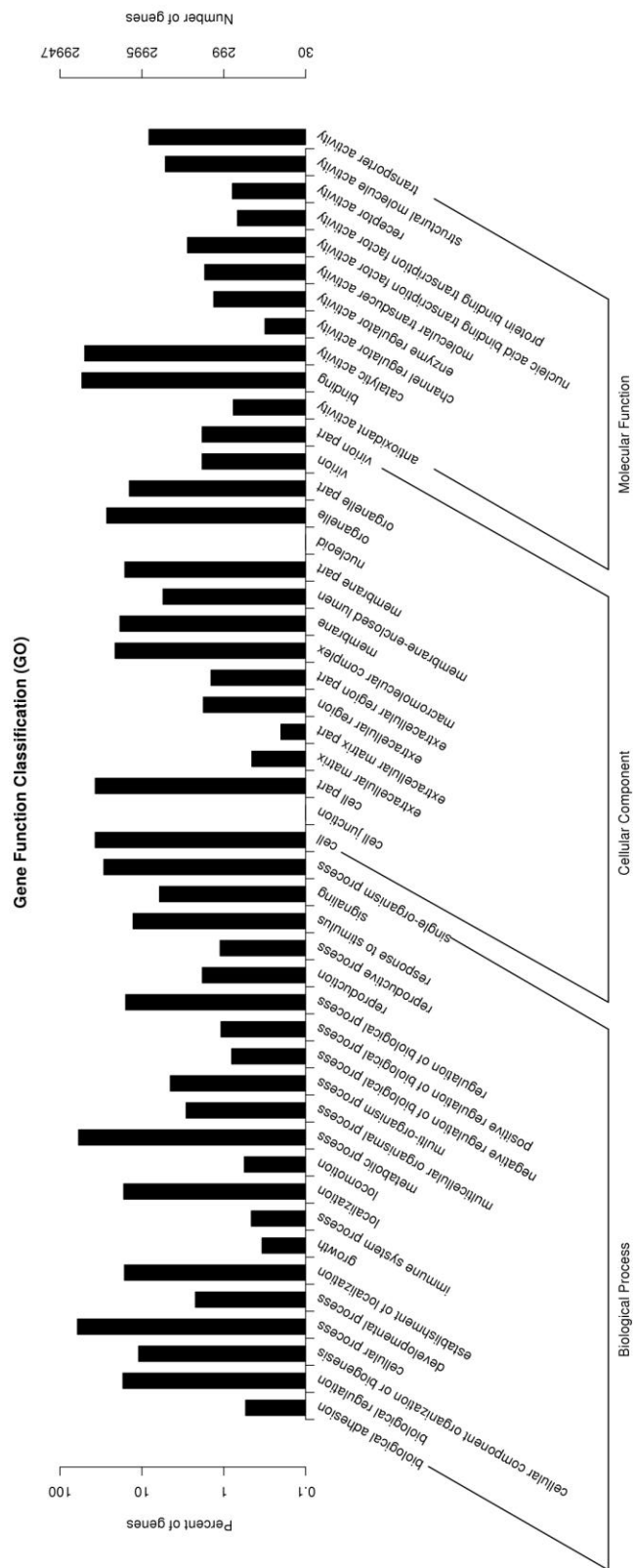

Supplement: Additional file 4: — The classification of the GOs. (PDF 205 kb) [file 12864_2016_3182_MOESM4_ESM.pdf]

Figure S2 The information of gene function classification (KEGG) analysis

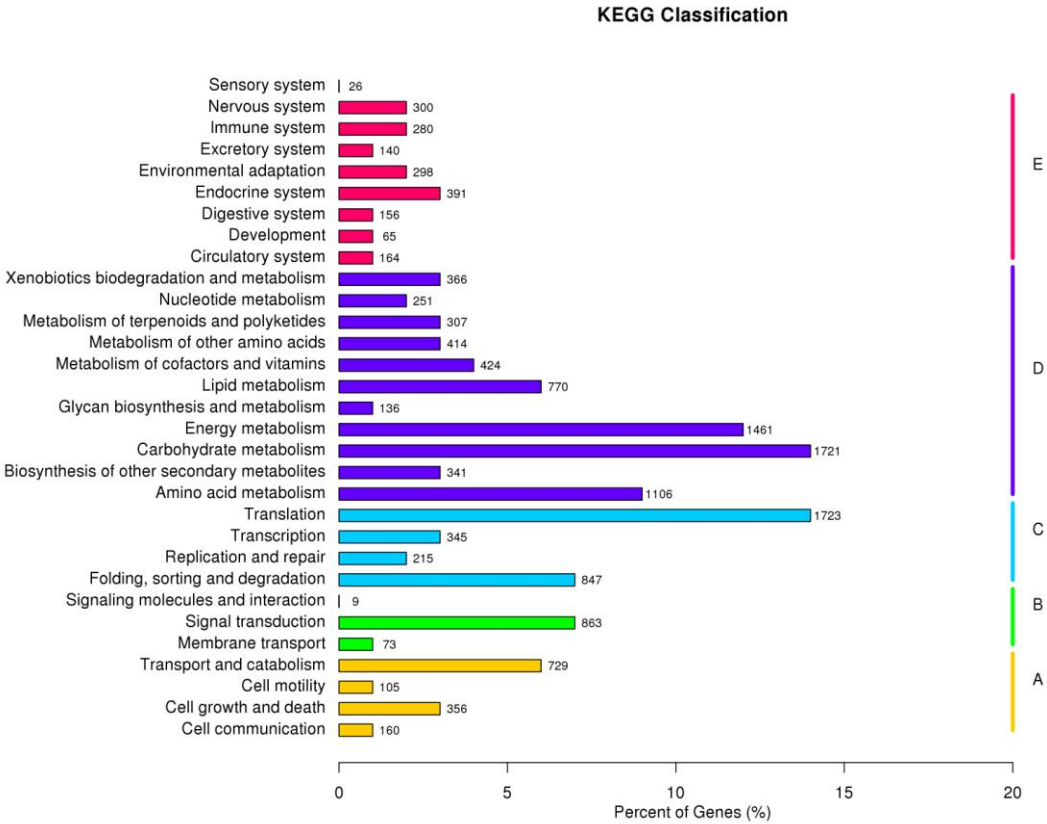

Supplement: Additional file 6: — The classification of the KEGGs. (PDF 181 kb) [file 12864_2016_3182_MOESM6_ESM.pdf]
